# Integrated bioinformatics analysis reveals upregulated extracellular matrix hub genes in pancreatic cancer: Implications for diagnosis, prognosis, immune infiltration, and therapeutic strategies

**DOI:** 10.1002/cnr2.2059

**Published:** 2024-04-19

**Authors:** Md Roman Mogal, Jasmin Akter Jame, Md Sohel, Md Mozibullah, Md Rashel Mahmod, Asadullah Junayed, Newton Kar, Lubatul Arbia, Abdullah Al Mamun, Md Asaduzzaman Sikder

**Affiliations:** ^1^ Department of Biochemistry and Molecular Biology Mawlana Bhashani Science and Technology University Tangail Bangladesh; ^2^ Department of Biochemistry and Molecular Biology Primeasia University Dhaka Bangladesh

**Keywords:** collagen proteins, extracellular matrix, hub genes, immune cells infiltration, pancreatic cancer

## Abstract

**Background:**

Pancreatic cancer (PC) stands out as one of the most formidable malignancies and exhibits an exceptionally unfavorable clinical prognosis due to the absence of well‐defined diagnostic indicators and its tendency to develop resistance to therapeutic interventions. The primary objective of this present study was to identify extracellular matrix (ECM)‐related hub genes (HGs) and their corresponding molecular signatures, with the intent of potentially utilizing them as biomarkers for diagnostic, prognostic, and therapeutic applications.

**Methods:**

Three microarray datasets were sourced from the NCBI database to acquire upregulated differentially expressed genes (DEGs), while MatrisomeDB was employed for filtering ECM‐related genes. Subsequently, a protein–protein interaction (PPI) network was established using the STRING database. The created network was visually inspected through Cytoscape, and HGs were identified using the CytoHubba plugin tool. Furthermore, enrichment analysis, expression pattern analysis, clinicopathological correlation, survival analysis, immune cell infiltration analysis, and examination of chemical compounds were carried out using Enrichr, GEPIA2, ULCAN, Kaplan Meier plotter, TIMER2.0, and CTD web platforms, respectively. The diagnostic and prognostic significance of HGs was evaluated through the ROC curve analysis.

**Results:**

Ten genes associated with ECM functions were identified as HGs among 131 DEGs obtained from microarray datasets. Notably, the expression of these HGs exhibited significantly (*p* < 0.05) higher in PC, demonstrating a clear association with tumor advancement. Remarkably, higher expression levels of these HGs were inversely correlated with the likelihood of patient survival. Moreover, ROC curve analysis revealed that identified HGs are promising biomarkers for both diagnostic (AUC > 0.75) and prognostic (AUC > 0.64) purposes. Furthermore, we observed a positive correlation between immune cell infiltration and the expression of most HGs. Lastly, our study identified nine compounds with significant interaction profiles that could potentially act as effective chemical agents targeting the identified HGs.

**Conclusion:**

Taken together, our findings suggest that COL1A1, KRT19, MMP1, COL11A1, SDC1, ITGA2, COL1A2, POSTN, FN1, and COL5A1 hold promise as innovative biomarkers for both the diagnosis and prognosis of PC, and they present as prospective targets for therapeutic interventions aimed at impeding the progression PC.

## 1 INTRODUCTION

The pancreas, positioned as a digestive gland in the posterior abdominal region, concurrently serves exocrine and endocrine roles in the enzymatic breakdown of dietary substances and the homeostatic regulation of blood glucose levels. However, a malignant cancerous tumor develops when healthy pancreas cells fail to function properly and grow out of control. The exact reason for pancreatic cancer (PC) is unknown, but many factors, including pancreatitis, smoking, obesity, family history of PC, gallbladder illness, alcohol drinking, dietary factors, and infection are the risk factors for PC.^1^ PC is one of the worst cancers and the seventh biggest cause of cancer‐related deaths, with an anticipated 458 918 new PC patients and 432 242 fatalities worldwide in 2018.^2^ Studies have shown that higher levels of physical activity can reduce the risk of developing PC.^3^, ^4^ Therefore, it can be inferred that leading a sedentary lifestyle may elevate the likelihood of developing PC. In developed nations, the occurrence of PC is observed to be three to four times greater when juxtaposed with its incidence in developing and underdeveloped countries.^5^ American Cancer Society predicts that about 64 050 people will have PC, and 50 550 people will die from PC in 2023.^6^ By 2030, it is anticipated that PC will be the second‐leading cause of cancer‐related death in Western Europe and North America by overtaking prostate, breast, and colorectal cancers.^7^, ^8^

PC poses a significant risk to human health due to its subtle onset, quick progression, and unfavorable prognosis.^9^ PC exhibits a comparatively lower frequency of occurrence yet demonstrates a heightened fatality rate in contrast to other malignancies such as breast, lung, colorectal, and gastric cancer. More than half of PC‐related deaths were recorded in developed countries, with 226 272 people deaths.^10^ Globally, the mortality rates associated with PC exhibit an escalation with advancing age, with a marginal predilection towards higher rates in males compared to females.^2^ The majority of PC fatalities, nearly 90%, manifest after the age of 55, revealing an augmented mortality risk as individuals progress in age.^2^ The overall survival of PC is very poor; just 24% of people survive 1 year, and 9% survive 5 years after diagnosis with PC.^10^ The average 5‐year survival rate for PC patients in the USA is 11%.^11^

Currently, the available PC treatments are surgery, chemotherapy, immunotherapy, and radiotherapy. Surgery is the best treatment option and is particularly effective for PC in its early stages. However, owing to the concealed and latent characteristics inherent to PC, it is commonly identified at an advanced stage, with 80%–90% of patients presenting unresectable tumors at the time of diagnosis. The National Cancer Institute in the USA showed that for patients who were diagnosed with PC at the primary stage, the 5‐year survival rate was 32%. In addition, The 5‐year survival rate for PC at stage II and stage III was 12% and 3%, respectively.^10^ Therefore, early PC diagnosis can lead to better patient outcomes. The available diagnostic tools are MRI, abdominal ultrasonography, endoscopic ultrasound‐guided fine‐needle aspiration, and tri‐phasic pancreatic‐protocol CT.^12^, ^13^ Still, those are not reliable enough to be considered clinically practicality for diagnosing PC at an early stage.^14^

Additionally, the USA Food and Drug Administration (FDA) approved carbohydrate antigen 19–9 (CA19‐9) as a serological marker which is helpful in the diagnosis and prognosis of PC. However, due to its limited sensitivity and specificity, this marker is not thought to be the most efficient.^15^ Moreover, lacking fucosyltransferase activity in individuals with homozygous mutations in the FUT3 gene, CA19‐9 is not observed in 5%–10% of patients.^8^ On the other hand, PC treatment is unsatisfactory due to multiple drug resistance. Drug resistance is a multifactorial aspect, including lacking bioavailability of the drugs in tumor cells, activating alternative pathways, aberration of drug metabolism, tumor microenvironment, immune cells, and stromal compartment.^16^, ^17^ From the previous decade, gemcitabine has been a mainstay of first‐line therapy for advanced PC. However, gemcitabine shows more resistance to PC cells than other drugs, severely limiting PC treatment effectiveness.^18^ Therefore, finding relevant biomarkers that could be used for diagnosis, prognostic, and therapeutic purposes is imperative.

**FIGURE 1:**
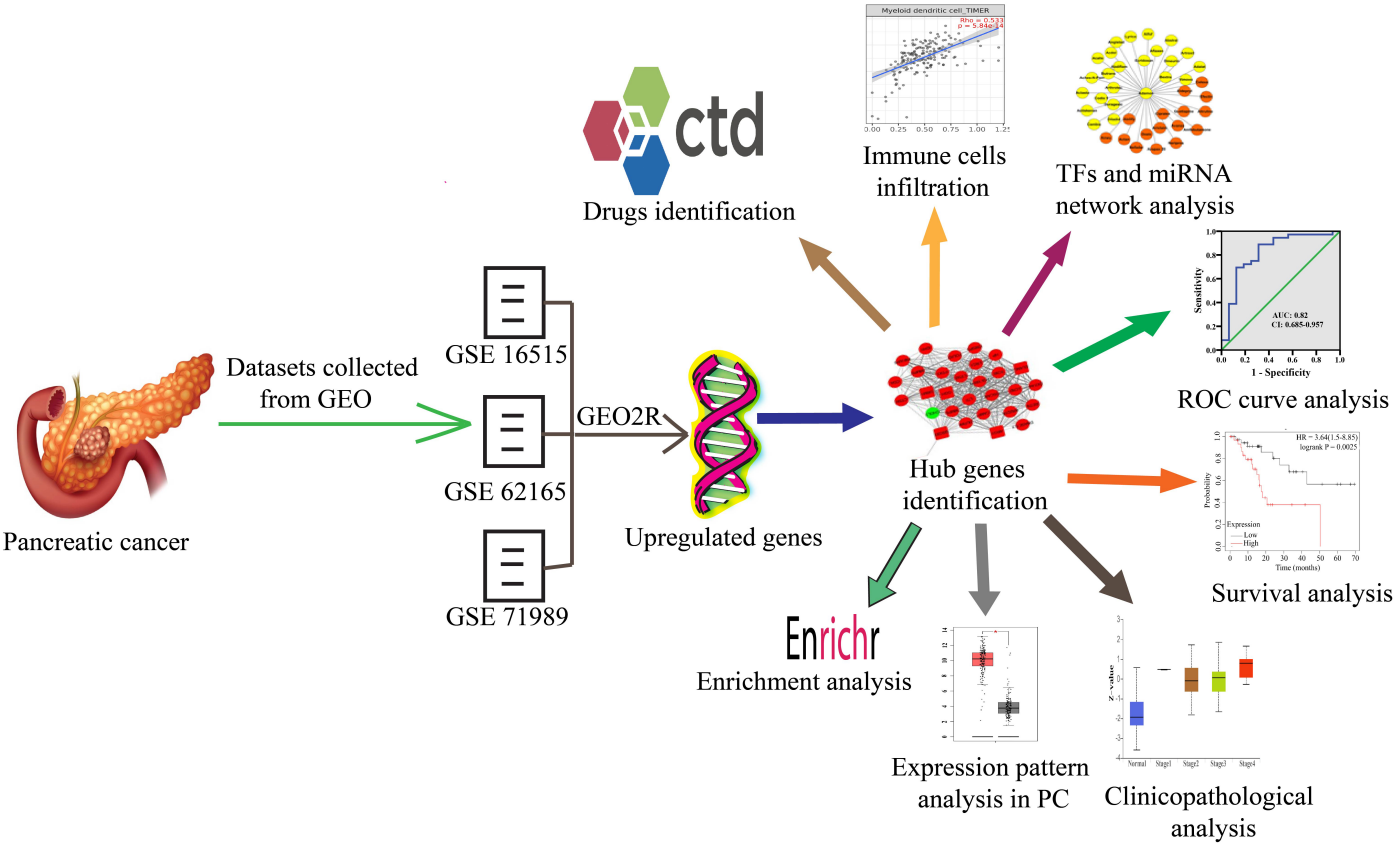
Schematic representation of the overall workflow of this study.

An extracellular matrix (ECM) is a non‐cellular, complex, and dynamic network of macromolecules that provide structural and biochemical support to cells. In contrast to the extracellular matrix of normal tissue, the extracellular matrix of tumors contains a greater concentration of collagen and hyaluronic acid. The ECM plays an important role in cancer progression by influencing cell behavior as well as the microenvironment within the tumor.^19^, ^20^ Crosslinking of the ECM by cancer and stromal cells leads to matrix stiffening, which activates specific transcription factors like Yes‐associated protein (YAP)/transcriptional coactivator with PDZ‐binding motif (TAZ), β‐catenin, and nuclear factor kappa B (NF‐κB).^21^ These transcription factors are responsive to changes in matrix stiffness and play a role in fostering malignant characteristics within cancer and stromal cells, including cancer‐associated fibroblasts. In addition, the ECM modulates cell mechanosensation and influences tumor cell invasion and metastatic growth. The overexpression of fibroblast activation protein (FAP) in fibroblasts results in the production of an ECM that enhances the velocity and directionality of PC cell invasion.^22^ A study published by Vaquero et al indicated that ECM proteins, particularly laminin and fibronectin, inhibit mitochondrial dysfunction and caspase activity in PC cells.^23^ Studies have demonstrated that ECM proteins can protect cancer cells from the cell death‐inducing effects of chemotherapy drugs.^24^, ^25^ In addition, the formation of the ECM in solid tumors can hinder the effectiveness of drugs by impeding the infiltration of therapeutic agents. The ECM governs the activity of immune cells, including tumor‐associated macrophage, dendritic cells, and T cells, influencing their performance and fostering an immunosuppressive tumor microenvironment.^26^, ^27^ More precisely, the activation of ECM genes in fibroblasts associated with cancer can result in the ability to evade the immune system and become resistant to immunotherapy. These findings emphasize the significance of taking into account the ECM to identify the novel biomarkers. Identifying ECM‐related HGs in PC holds immense promise for developing new treatment strategies, improving PC patient outcomes, and potentially even preventing PC progression. However, the specific genes and proteins that could potentially function as prognostic and therapeutic biomarkers for PC remain unknown. The objective of this study was to identify ECM‐related biomarkers for PC through integrated Bioinformatics approaches. We proposed that certain genes upregulated in relation to the ECM could potentially function as biomarkers for PC. The overall workflow for this study is depicted in Figure 1.

## 2 MATERIALS AND METHODS

### 2.1 Data collection and processing

Three gene expression datasets (GSE16515, GSE62165, and GSE71989) were retrieved from the GEO database (https://www.ncbi.nlm.nih.gov/gds) by typing the keywords ‘pancreatic cancer’ and selecting “*Homo sapiens*,” “expression profiling by array.” GPL570 (Affymetrix Human Genome U133 Plus 2.0 Array) platform was utilized for both the GSE16515 and GSE71989 datasets. On the other hand, for the GSE62165 dataset, the GPL13667 (Affymetrix Human Genome U219 Array) platform was adopted. The GSE16515 contained a total of 52 samples of 36 pancreatic tumor samples and 16 normal samples. The GSE62165 datasets contained 118 pancreatic ductal adenocarcinomas and 13 normal samples, and the GSE71989 datasets comprised 14 pancreatic ductal adenocarcinomas and 8 normal samples.

### 2.2 Identification of commonly upregulated ECM genes

GEO2R (https://www.ncbi.nlm.nih.gov/geo/geo2r/) is a dynamic web platform having GEOquery and limma packages from the Bioconductor project that is exploited to find DEGs by comparing different groups of samples in a GEO series under different experimental conditions.^28^ GEO2R was employed to determine the DEGs between pancreatic tumor tissues and normal tissues. Benjamini–Hochberg was utilized to maintain the false discovery rate.^29^ Subsequently, the DEGs from each dataset were retrieved in table format using GEO2R and then imported into RStudio for subsequent analysis. In the pursuit of upregulated genes, datasets were refined based on criteria, specifically adjusted *p*‐value <0.01 and Log2‐fold change >2. A Venn diagram was made to graphically visualize the upregulated genes among three datasets by using the Bioinformatics and Evolutionary Genomics web platform (http://bioinformatics.psb.ugent.be/webtools/Venn/). MatrisomeDB 2.0 database (https://matrisomedb.org/) was utilized to filter genes associated with the ECM. MatrisomeDB 2.0 is a comprehensive resource on ECM and ECM‐associated genes and proteins that aggregates silico and experimental data from various tissues and tumors.^30^

### 2.3 Network construction and HGs identification

The construction of the protein–protein interaction (PPI) network for the ECM upregulated genes was executed utilizing the STRING database (https://string-db.org/). Subsequently, the PPI network was displayed using the Cytoscape software (version 3.9.1). Cytoscape is open‐source software equipped with diverse plugin tools, that facilitate the visualization of molecular interaction networks and biological pathways.^31^ The identification of hub genes (HGs) within the network was accomplished using a Cytoscape plugin named CytoHubba, which employs 11 topological analysis methods. Among them, Maximal Clique Centrality (MCC) and Degree topological methods were applied in this study.

### 2.4 Enrichment analysis of HGs

Enrichment analysis is a well‐established method for analyzing the collective behavior of genes in terms of health and disease conditions.^32^ The signaling pathways and associated gene ontologies of hub genes (HGs) were acquired through Enrichr (https://maayanlab.cloud/Enrichr/), an accessible online enrichment analysis tool that provides user‐friendly functionality and diverse visualization summaries for gene list analyses.^33^, ^34^ KEGG 2021 Human, BioPlanet 2019, WikiPathway 2021 Human, and Reactome 2022 databases were adopted for pathways analysis. Moreover, gene ontologies were elucidated through GO Biological Process 2021, GO Molecular Process 2021, and GO Cellular 2021.

### 2.5 mRNA expression of HGs in PC

GEPIA2 (http://gepia2.cancer-pku.cn/#degenes) was adopted to investigate the mRNA expression of HGs for PC. GEPIA2 (Gene Expression Profiling Interactive Analysis 2) systematically evaluates gene expression patterns across tumor and normal tissues, drawing data from the TCGA and GTEx databases.^35^ The cutoff |Log2FC| value and *p*‐value were 1 and 0.01, respectively. Moreover, the HG expression in PC compared to their normal counterpart was obtained from the OncoDB web platform. OncoDB is an online platform to examine aberrant patterns in gene expression and viral infection associated with cancer clinical characteristics.^36^

### 2.6 Clinicopathological analysis of HGs

The expression pattern of 10 HGs in different cancer stages and tumor grades was investigated based on the CPTAC database through the ULCAN (http://ualcan.path.uab.edu/) web platform. CPTAC is a National Cancer Institute program that uses extensive mass spectrometry‐based proteomics to hasten our understanding of the molecular causes of cancer.^37^ For observing HGs expression, pancreatic adenocarcinoma datasets were selected from the CPTAC database.

### 2.7 Mutation and methylation status analysis

The mutation frequency of HGs was determined by adopting cBioPortal (https://www.cbioportal.org/). cBioPortal is a web‐based application that aids users in executing cohort analysis of different biological and clinically relevant hypotheses.^38^ In this study, 11 datasets with 1347 samples were selected from cBioPortal. In addition, promoter methylation is an important epigenetic aspect that adds a methyl group to the promoter region of genes and regulates gene expression by engaging proteins involved in gene repression. The promoter methylation of HGs was analyzed on TCGA data from the ULCAN web platform. The beta value in DNA methylation is calculated considering the intensities of both methylated and unmethylated signals at a specific CpG site. The equation is *β* = *M*/(*M* + *U* + *α*). *M* = methylated signal, *U* = unmethylated signal, and *α* is a small constant offset, typically set to 100.

### 2.8 Survival analysis of HGs

To examine the correlation between the expression levels of HGs and the survival outcomes of PC patients, the study utilized the Kaplan–Meier plotter (https://kmplot.com/analysis/), an online platform designed for assessing the clinical implications of gene expression.^39^ Here, we assessed the survival probability of PC patients based on overall survival (OS) and relapse‐free survival (RFS). Moreover, the data were divided at the median, and analysis was executed by selecting pancreatic ductal adenocarcinoma from Pan‐cancer RNA‐seq. A *p* < 0.05 was considered significant.

### 2.9 Verification of diagnostic and prognostic values by ROC curve analysis

To assess the diagnostic and prognostic significance of HGs, the study employed receiver operating characteristic (ROC) analysis based on their expression levels. Here, we obtained survival data from TCGA‐PAAD via the OncoLnc (http://www.oncolnc.org/) platform, and gene expression data (GSE16515) were collected from the GEO database. The ROC curves were drawn, and areas under the curve (AUC) for corresponding HGs were estimated using the Statistical Packages for Social Sciences (SPSS for Windows, version 20, IBM Corp., Armonk, New York, USA) software. Additionally, we bolstered our ROC curve findings by acquiring supplementary expression and survival data from the GSE183795 dataset via the R2 genomics (https://hgserver1.amc.nl/cgi-bin/r2/main.cgi) platform. The ROC curve was generated using SRplot (https://www.bioinformatics.com.cn/srplot).

### 2.10 Immune infiltrates analysis of HGs

The relationship between HGs and immune infiltration was examined by Tumor Immune Estimation Resource (TIMER 2.0, timer.comp-genomics.org/). This user‐friendly web tool employs six advanced algorithms for comprehensive immune infiltration analysis across various cancers.^40^ The research specifically examined the correlation between HGs' expression and the infiltration of T cell CD8+, neutrophils, dendritic cells, macrophages, and natural killer cells. To further elucidate the link between HGs' expression and immune infiltration in PC, the study employed the TISIDB platform (http://cis.hku.hk/TISIDB/).

### 2.11 TFs and miRNAs associated with HGs

Transcription factors (TFs) are proteins that regulate how genetic information is translated from DNA to mRNA. Short non‐coding RNAs called miRNAs control gene expression at the post‐transcriptional level. The TFs and miRNAs networks were constructed by utilizing NetworkAnalyst (https://www.networkanalyst.ca/), a sophisticated web application that is used to interpret gene expression data and generate networks.^41^ For making TFs and miRNAs networks, data were collected from JASPAR and TarBase databases, respectively. Additionally, the study employed the ULCAN web tool to discern the expression patterns of highly associated miRNAs with HGs.

### 2.12 Screening of chemical compounds for HGs in PC

The Comparative Toxicogenomics Database (CTD, https://ctdbase.org) is a cutting‐edge digital platform that links toxicological data for genes, diseases, phenotypes, chemicals, and exposures.^42^ The CTD (last visited on December 20, 2023) database was used in this study to obtain the interacted chemical compounds with HGs. The name of HGs was imported in the search box, and different compounds associated with HGs were obtained. Subsequently, from the huge number of combinations, desired compounds were sorted out by selecting “decreased gene expression” and “high interaction value >2.”

## 3 RESULTS

### 3.1 Screening upregulated genes in the microarray datasets

**FIGURE 2:**
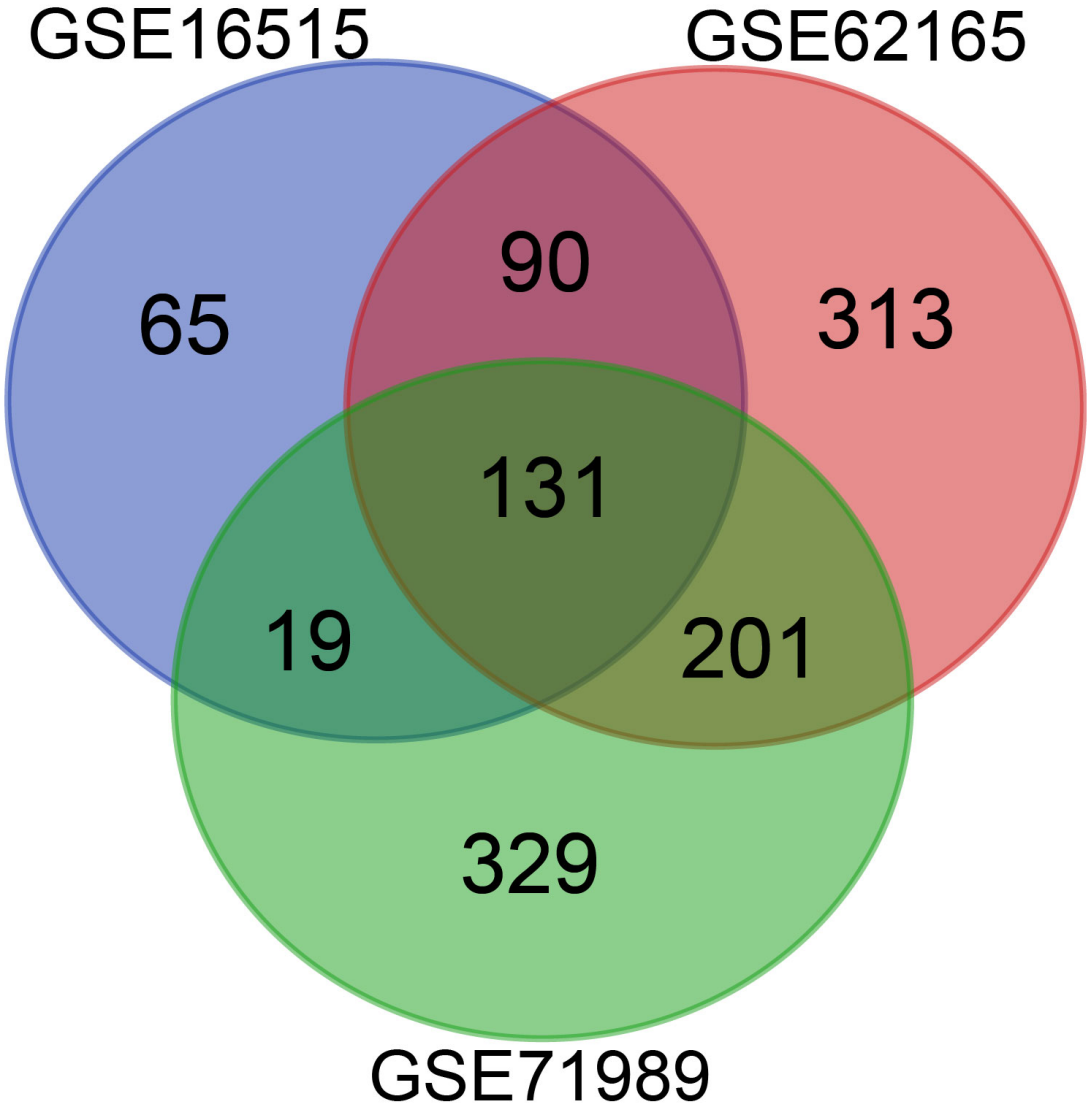
A Venn diagram of differentially expressed upregulated genes. One hundred and thirty‐one common upregulated genes were identified from GSE16515, GSE62165, and GSE71989 datasets.

In this research endeavor, we investigated ECM‐related HGs in PC through the analysis of three distinct microarray datasets. Within the GSE16515 dataset, we identified 305 upregulated genes, while the GSE62165 dataset showcased 735, and the GSE71989 dataset revealed 680. An intersection among these three datasets contained 131 common upregulated genes, as presented by a Venn diagram (Figure 2). After meticulous filtration of the MatrisomeDB database, a total of 87 genes associated with the ECM were identified.

### 3.2 Network construction and HGs selection

**FIGURE 3:**
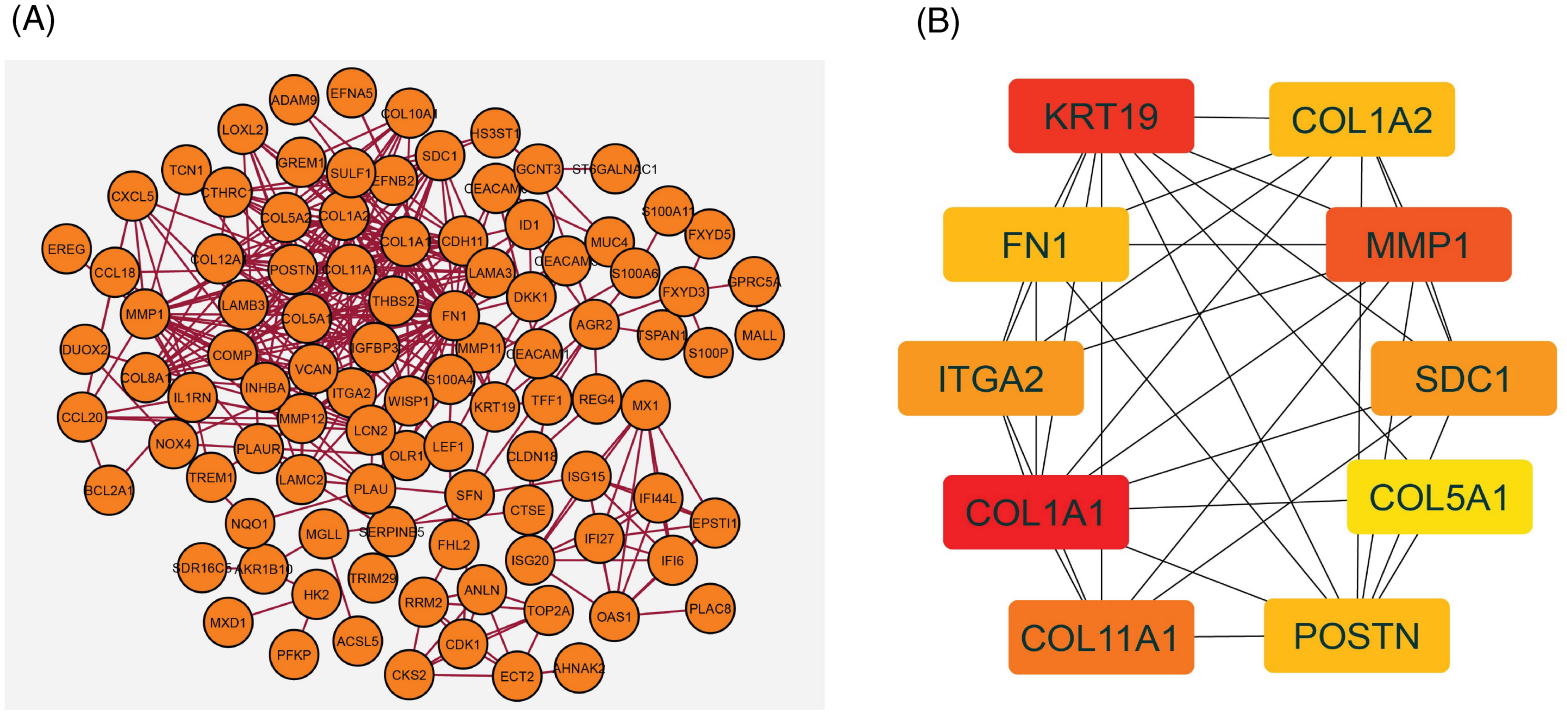
(A) Protein–protein interaction network consisting of 99 nodes and 344 edges. (B) Hub genes network with 10 hub genes.

Network analysis is integral to system biology, providing insightful knowledge about gene sets without studying individual genes.^32^, ^43^ The corresponding PPI network for upregulated genes was acquired from the STRING database and displayed by Cytoscape software. The PPI network comprised 99 nodes and 344 edges (Figure 3A). From this PPI network, 10 genes, namely COL1A1, KRT19, MMP1, COL11A1, SDC1, ITGA2, COL1A2, POSTN, FN1, and COL5A1, were identified as HGs, as illustrated in Figure 3B.

### 3.3 Functional enrichment analysis

**FIGURE 4:**
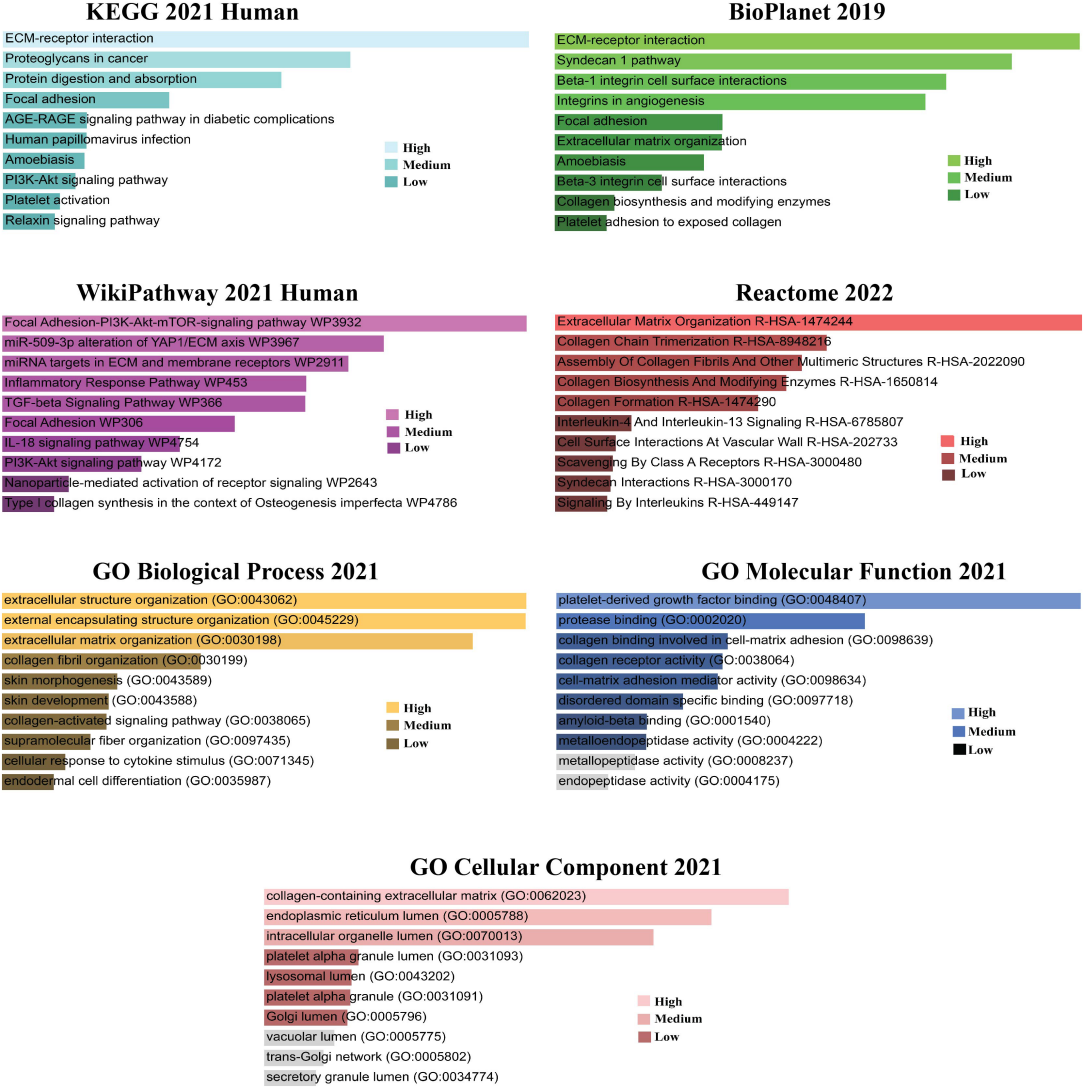
Signaling pathways (KEGG, BioPlanet, WikiPathway and Reactome) and gene ontologies (biological process, molecular function, and cellular component) for hub genes based on combined score.

Four databases were explored to find significantly enriched pathways associated with HGs, as shown in Figure 4. The KEGG 2021 Human revealed the pathways related to ECM interaction, proteoglycan in cancer, focal adhesion PI3K‐Akt, and relaxin signaling pathway. In addition, for BioPlanet 2019, the significant pathways included ECM‐receptor interaction, syndecan 1 pathway, beta‐1 integrin cell surface interactions, focal adhesion, extracellular matrix organization, collagen biosynthesis, and platelet adhesion. Moreover, the prominent pathways for the WikiPathway 2021 Human were PI3K‐Akt–mTOR signaling, miR‐509‐3p alteration, inflammatory response, TGF‐beta signaling, IL‐18 signaling, and focal adhesion. Furthermore, in the analysis of Reactome 2022, HGs were predominantly enriched in the extracellular matrix organization, collagen chain termination and biosynthesis, interleukin‐4 and interleukin‐13 signaling, cell surface interaction, and syndecan interactions. The key gene ontologies for the HGs were as follows: extracellular structure and matrix organization, collagen‐related activities, platelet‐derived growth factor, protease binding, and endoplasmic reticulum lumen (Figure 4).

### 3.4 HGs expression pattern in PC

**FIGURE 5:**
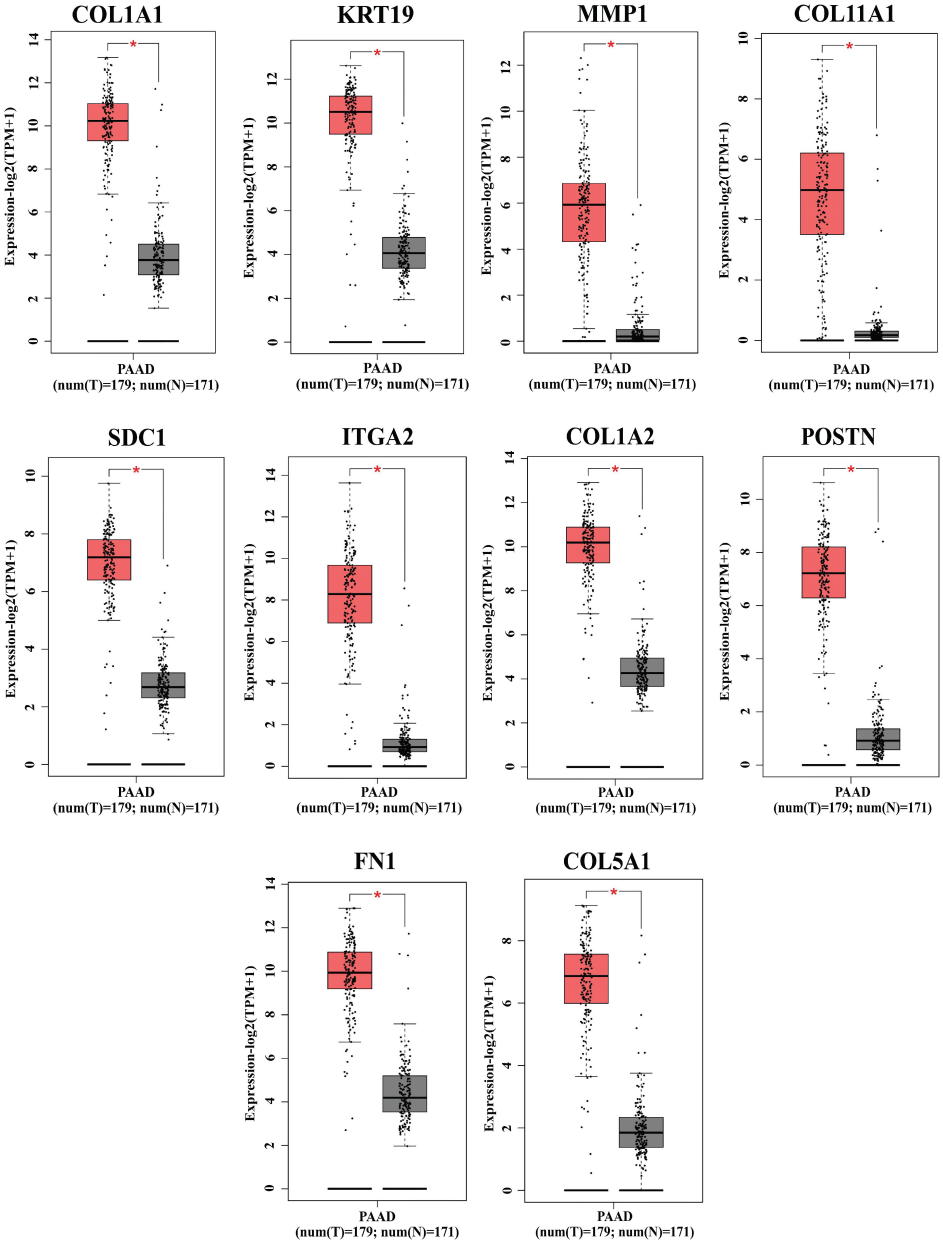
Comparison of the hub genes expression in PC and normal tissue. The boxplot shows the hub gene expression in normal tissue (right) and PC (left) (*indicates *p* ≤ 0.05).

Utilizing the GEPIA2 web platform, our investigation uncovered significant overexpression of HGs such as COL1A1, KRT19, MMP1, COL11A1, SDC1, ITGA2, COL1A2, POSTN, FN1, and COL5A1 in PC compared to normal tissue (Figure 5). Similarly, analysis through the OncoDB web tool showed a higher expression of HGs in PC compared to normal (Figure S1). These findings suggest a significant association between the upregulation of HGs and PC progression.

### 3.5 HGs expression based on cancer stages and tumor grades

**FIGURE 6:**
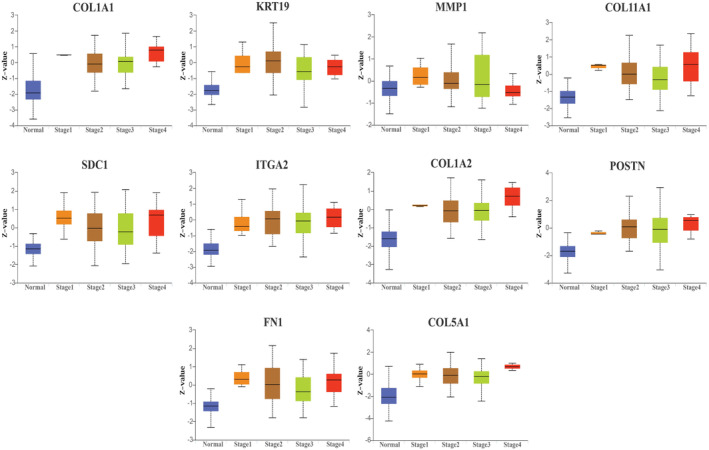
Protein expression of hub genes in PC based on cancer stages.

Upon conducting clinicopathological analysis, it was observed that the expression of HGs was notably elevated across different cancer stages when compared to normal groups (Figure 6). Similarly, high expression of HGs was also found in various tumor grades (Figure S2). The intensity of hub gene expression exhibited an incremental pattern corresponding to the advancement of cancer stages and tumor grades.

### 3.6 Determination of mutation and methylation status of HGs

**FIGURE 7:**
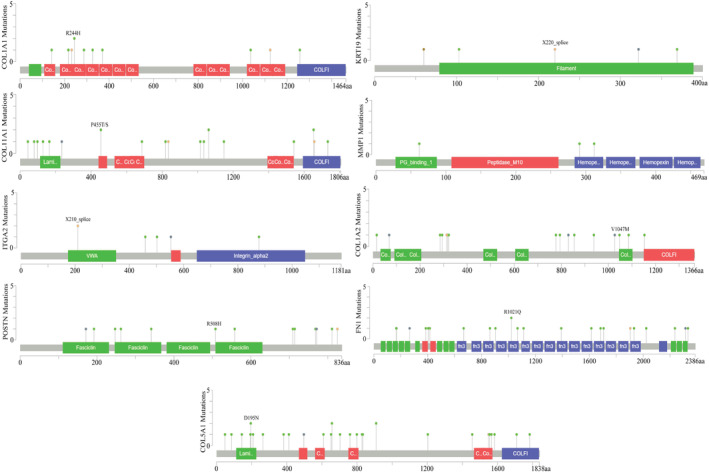
Genomic mutations determination of Hub genes in PC.

cBioPortal was employed to investigate the mutation frequency of HGs. The somatic mutation frequency of COL1A1, KRT19, MMP1, COL11A1, ITGA2, COL1A2, POSTN, FN1, and COL5A1 were 0.9%, 0.4%, 0.3%, 1.8%, 0.5%, 1.3%, 1.0%, 1.9%, and 1.7%, respectively (Figure 7). On the other hand, from methylation analysis, we observed that the promoter region of HGs such as KRT19, MMP1, COL11A1, SDC1, FN1, and COL5A1 is significantly methylated in normal tissue than in tumor tissue. However, other HGs, especially collagen‐related genes, including COL1A1, COL1A2, ITGA2, and POSTN revealed slightly higher methylation in tumor tissue than in normal tissue (Figure S3).

### 3.7 Survival analysis of HGs in PC

**FIGURE 8:**
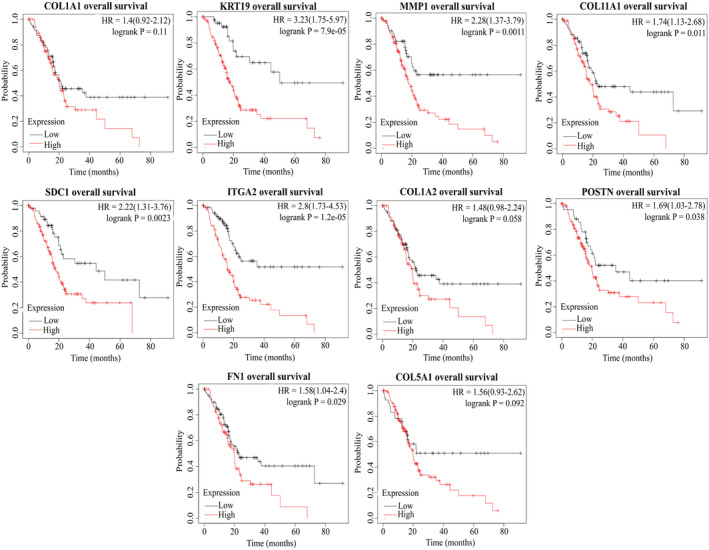
Correlation between hub genes expression and overall survival probability of PC patients. Red lines indicate hub gene overexpression, and blue lines indicate low hub gene expression.

**FIGURE 9:**
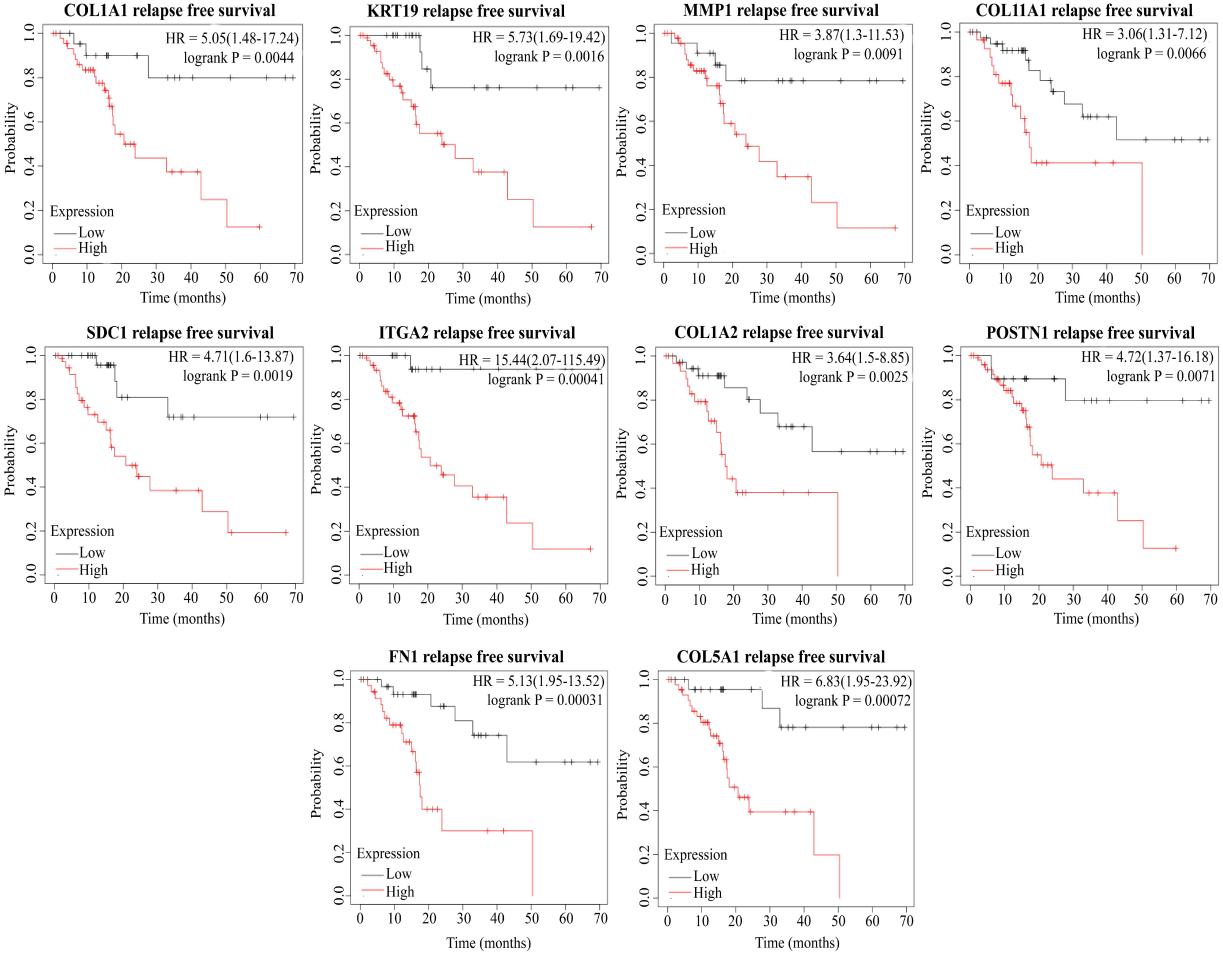
Correlation between hub genes expression and relapse‐free survival probability of PC patients. Red lines indicate hub gene overexpression, and blue lines indicate low hub gene expression.

To ascertain the impact of HGs upregulation on the survival probability of PC patients, we conducted a comprehensive survival analysis. The findings revealed a compelling association between elevated HGs expression and diminished overall survival (OS) as well as recurrence‐free survival (RFS) probabilities. The hazard ratio (HR) of OS for COL1A1, KRT19, MMP1, COL11A1, ITGA2, COL1A2, POSTN, FN1, and COL5A1 were 1.4, 3.23, 2.28, 1.74, 2.8, 1.48, 1.69, 1.58, and 1.56, respectively (Figure 8). Furthermore, RFS also showed lower survival probability according to higher expression of HGs, COL1A1 (HR = 5.05), KRT19 (HR = 5.73), MMP1 (HR = 3.86), COL11A1 (HR = 3.06), ITGA2 (HR = 15.44), COL1A2 (HR = 3.64), POSTN (HR = 4.72), FN1 (HR = 5.13), and COL5A1 (HR = 6.83) (Figure 9).

### 3.8 ROC curve analysis of the HGs

**FIGURE 10:**
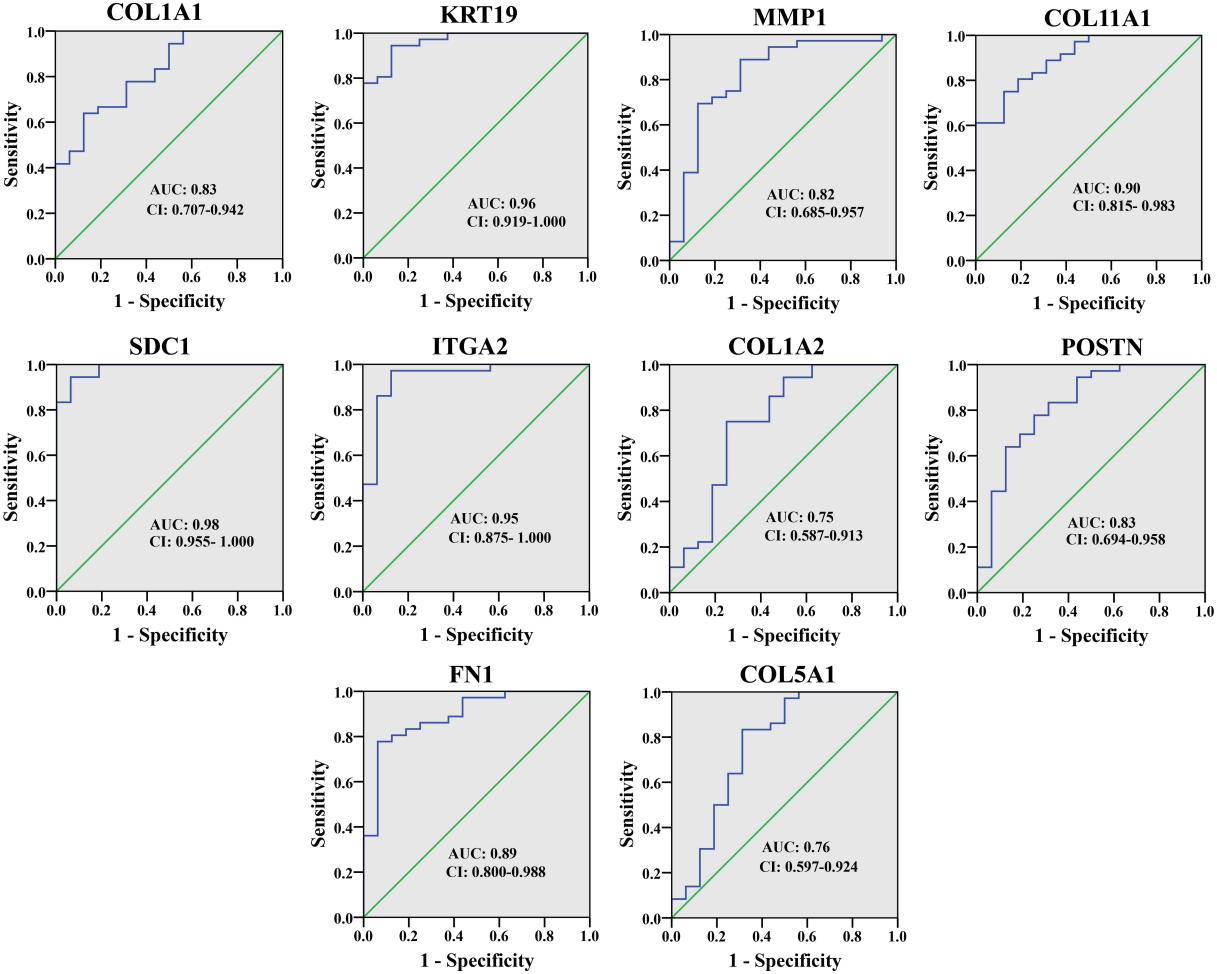
ROC curve analysis for determining hub genes as a diagnostic marker.

**FIGURE 11:**
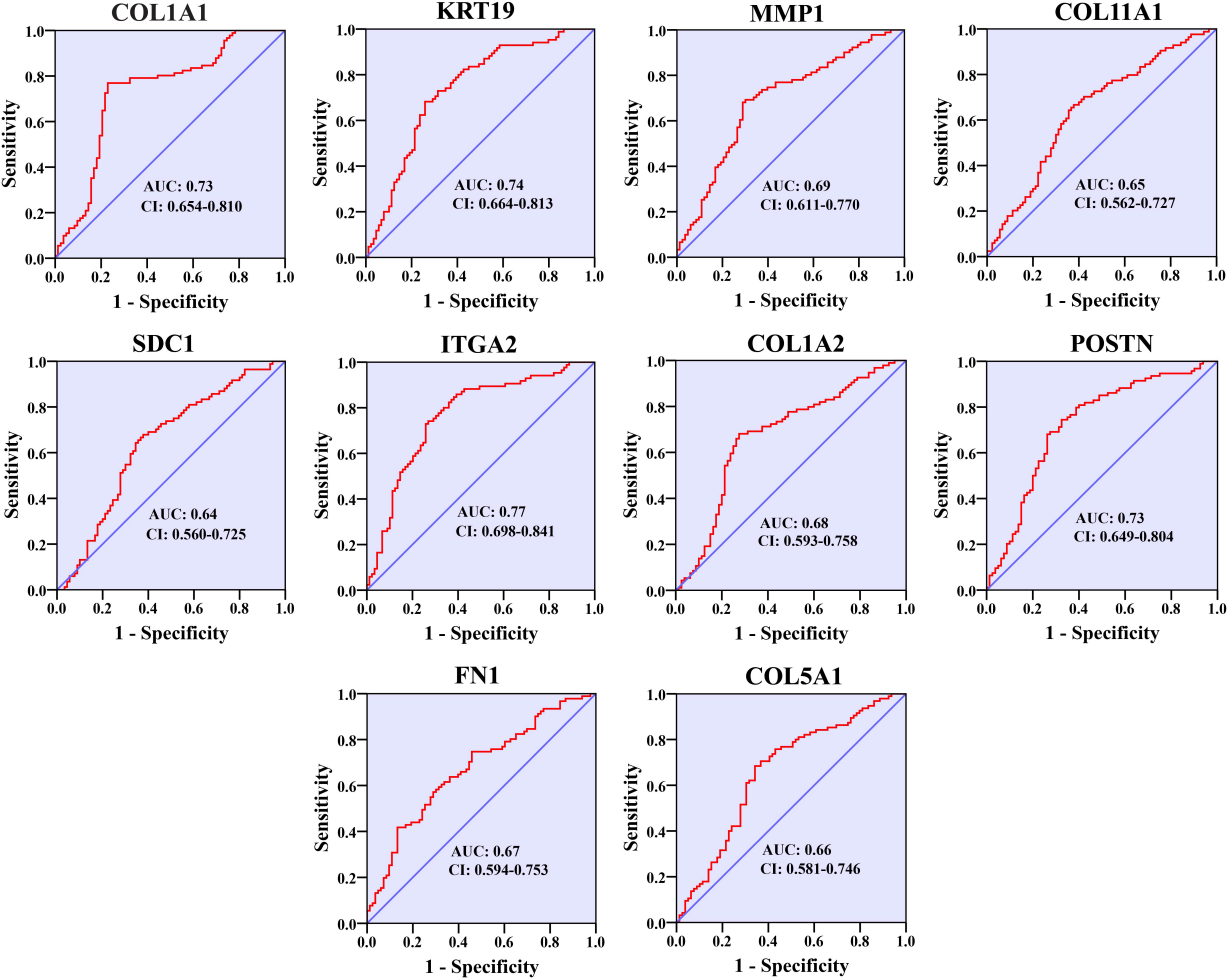
ROC curve analysis for determining hub genes as a prognostic marker.

ROC curve analysis was executed to determine the diagnostic significance of HGs in differentiating between normal pancreas tissues and PC tissues. As shown in Figure 10, for GSE16515 dataset, the AUC was 0.83 for COL1A1, 0.96 for KRT19, 0.82 for MMP1, 0.90 for COL11A1, 0.98 for SDC1, 0.95 for ITGA2, 0.75 for COL1A2, 0.83 for POSTN, 0.89 for FN1 and 0.76 for COL5A1 (Figure 10). Furthermore, the AUC for the survival of the patients was 0.73, 0.74, 0.69, 0.65, 0.64, 0.77, 0.68, 0.72, 0.67, and 0.66 (Figure 11). Furthermore, we confirmed the validity of our ROC curve outcome through the utilization of expression and survival data from the GSE183795 dataset (Figures S5 and S6).

### 3.9 Immune infiltrates analysis of HGs

**FIGURE 12:**
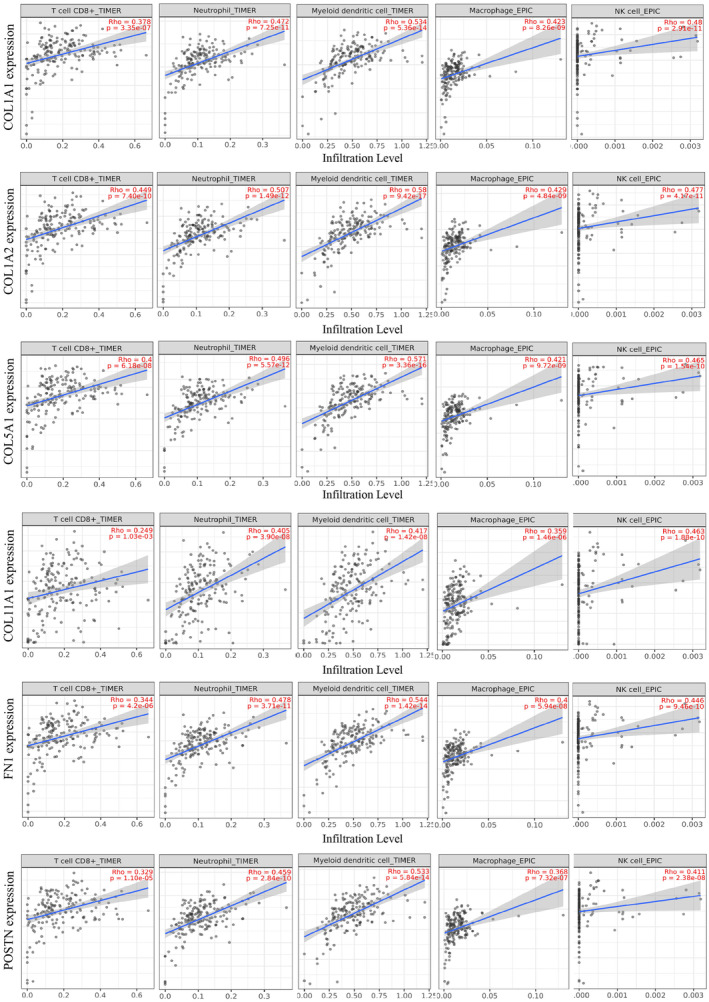
Correlation between six hub gene expressions and infiltration levels of immune cells in PC.

Immune infiltration is closely associated with the clinical outcomes of patients, and that can be used as a predictive tool for proper treatment in terms of chemotherapy and immune therapy. In our analysis, the expressions of six HGs such as COL1A1, COL1A2, COL11A1, COL5A1, POSTN, and FN1, are positively associated with immune infiltration of T‐cell CD8+, neutrophils, monocytes, macrophages, and dendritic cells (Figure 12). The rest of the four HGs, including KRT19, MMP1, SDC1, and ITGA2, did not show an association with immune infiltration. Additionally, immune infiltration analysis through TISIDB provided the same outcomes as TIMER 2.0 along with (Table S1).

### 3.10 TFs and miRNAs network analysis of HGs

**FIGURE 13:**
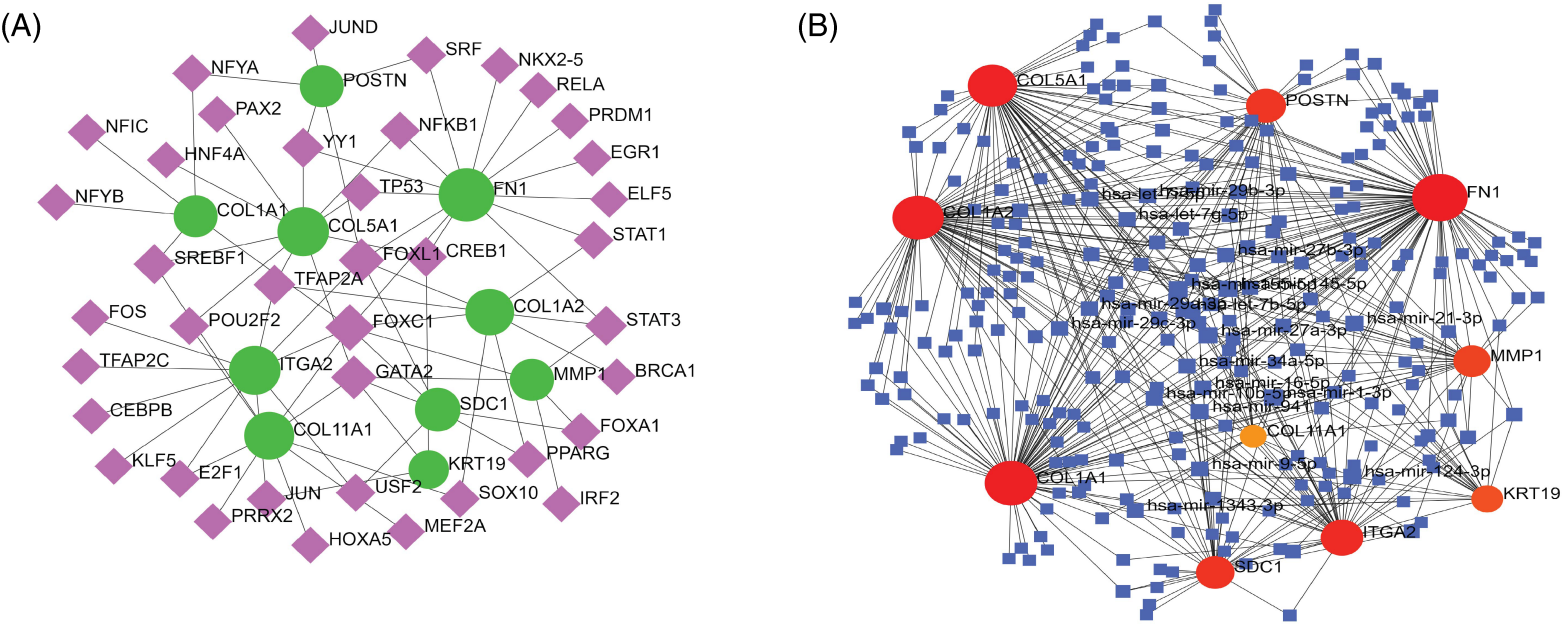
Interaction network of hub genes with TFs and miRNAs. (A) TF network constructed from the JASPAR database through the NetworkAnalyst platform. (B) miRNAs network constructed from the TarBase database using the NetworkAnalyst platform.

A TFs regulatory network with 49 interactions, 39 TFs, and 10 HGs was created using the NetworkAnalyst platform. The TFs that have high degree interaction are FOXC1, GATA2, FOXL1, CREB1, YY1, STAT3, TFAP2A, and SREBF1 (Figure 13A). On the other hand, the miRNAs network consisted of 279 nodes and 528 edges with three highly connected miRNAs (hsa‐mir‐145‐5p, hsa‐mir‐27a‐3p, and hsa‐mir‐124‐3p) (Figure 13B). In addition, expression analysis of three miRNAs revealed lower expression of miRNAs in PC compared to normal (Figure S4).

### 3.11 Identification of chemical compounds decreasing HGs expression in PC

Since our HGs are upregulated in PC, finding compounds that can decrease HGs expression in PC is imperative. We used the CTD database to search for prospective therapeutic substances that could lower PC patient's mRNA expression of the HGs (Table 1). In this aspect, we obtained nine chemical compounds: Resveratrol, Tetrachlorodibenzodioxin, JQ1, Quercetin, Tretinoin, Genistein, Ethinyl Estradiol, Progesterone, and Dexamethasone. The 3D structure chemical compound was retrieved from the PubChem database (Figure S7).

**TABLE 1:** Potential therapeutic compounds that can result in decreased expression of HGs.

Gene name	Chemical name	Interaction action	Reference count	Organism count
COL1A1	Resveratrol	Decrease expression	7	3
KRT19	Tetrachlorodibenzodioxin	Decrease expression	5	3
MMP1	(+)‐JQ1 compound, Quercetin	Decrease expression	3	1
CO11A1	Tretinoin, Progesterone	Decrease expression	2	2
SDC1	Tetrachlorodibenzodioxin	Decrease expression	9	2
ITGA2	Genistein	Decrease expression	2	2
COL1A2	Tetrachlorodibenzodioxin	Decrease expression	7	4
POSTN	Tetrachlorodibenzodioxin, Ethinyl Estradiol	Decrease expression	3	3
FN1	Tetrachlorodibenzodioxin	Decrease expression	9	3
COL5A1	Dexamethasone	Decrease expression	4	3

## 4 DISCUSSION

PC is considered a silent killer since it shows symptoms similar to other diseases, including ulcers, gastritis, and pancreatitis.^15^ Over the years, extensive studies have been executed to widen our understanding of PC pathogenesis and therapeutic strategies, but the clinical outcome of PC remains poor. Therefore, finding precise molecular biomarkers for PC progression and metastasis is crucial for diagnosing and treating PC patients. Bioinformatics has emerged as a prominent area in identifying disease‐causing genes, paving the way for more targeted and effective therapies. By utilizing, bioinformatics approaches, studies have identified PC‐associated genes and their molecular mechanisms.^44^, ^45^ This study analyzed three microarray datasets from the GEO database and obtained 131 common upregulated genes in PC. Afterward, a PPI network was constructed, and 10 genes were identified as HGs.

Enrichment analysis of HGs revealed signaling pathways and gene ontologies associated with cancer progression. The notable pathways from the KEGG 2021 database included ECM–receptor interaction, proteoglycans in cancer, protein digestion and absorption, and focal adhesion. The ECM–receptor interaction pathway has been reported to contribute a significant role in tumor shedding, movement, adhesion, degradation, and hyperplasia.^46^ Proteoglycans are polyhedric in nature, having the capability to interact with both ligands and receptors. They are the essential molecular effectors on the cell surface and conduct several functions in cancer progression and angiogenesis.^47^, ^48^ In addition, BioPlanet 2019 provided eminent pathways, including ECM‐receptor interaction, syndecan1 pathway, beta‐1 integrin cell surface interactions, and focal adhesion. It has been documented that the syndecan1 protein promotes carcinogenesis via the Wnt signaling pathway and is found in pancreatic, myeloma, myeloma, breast, and colorectal cancers.^49^, ^50^, ^51^, ^52^ The high level of syndecan1 protein is associated with cell proliferation, differentiation, and invasion of cancers.^53^ A study by Md Saimon et al. found that beta‐1 integrin protein stimulates the growth of pancreatic tumors by increasing the expression of Kindlin‐2 and TGF‐Receptor‐2.^54^ Moreover, Reactome 2022 showed extracellular matrix organization, collagen biosynthesis, modifying, chain trimerization, and assembly of collagen fibrils. Collagen family proteins are copiously found in the ECM environment. Besides their function in tissue organization and shaping, they have critical roles in tumorigeneses, cancer cell proliferation, cancer cell invasion, metastasis, cancer cell death resistance, and anti‐cancer immunity regulation.^55^, ^56^ Recent research has uncovered a protective role of collagen type 1 protein in PC. This protective function involves the inhibition of myeloid‐derived suppressor cells, which are known to dampen the anti‐tumor immune response.^57^ In these aspects, more research is required to ascertain the functions of collagen proteins in tissue‐dependent types of cancer.

The significant upregulation of HGs was observed in PC compared to normal tissue by GEPIA2. Specifically, the expression levels of four members of the collagen family, namely COL1A1, COL1A2, COL11A1, and COL5A1, exhibited an increase in PC tissues. Collagen proteins provide structural support to tissues and interact with cells to proliferate, migrate, and differentiate.^58^ COL1A1, COL1A2, COL11A1, and COL5A1 have been reported to regulate several cancers, including colorectal cancer, esophageal cancer, hepatocellular carcinoma, renal cell carcinoma, breast cancer, and gliomas.^59^, ^60^, ^61^, ^62^, ^63^ The ECM‐related genes, including MMP1, POSTN, and FN1, have shown higher expression in breast cancer, non‐small lung cancer, bladder cancer, and gastric cancer.^64^, ^65^, ^66^ On the other hand, keratin 19 (KRT19) is a small cytoplasmic intermediate filament, lacking tail domain among other cytokeratin, that maintains the structural rigidity of cells.^67^ A study on breast cancer demonstrated that increased KRT19 expression was correlated with breast cancer invasiveness.^68^ Moreover, syndecan1 (SDC1) is an integral membrane protein that is essential for cell proliferation and migration. SDC1 expression levels have been found to be greater in breast cancer and to be associated with a worse prognosis for breast cancer patients.^69^ Furthermore, ITGA2 encodes the alpha subunit of collagen receptors and binds with the beta subunit, which mediates cell adhesions to the extracellular matrix.^70^ Along with other types of cancers, upregulation of ITGA2 was reported in PC, which is responsible for metastasis and chemoresistance of PC.^71^ The clinicopathological analysis showed most of our HGs increased their expression level along with tumor stages and grades. This finding demonstrates a link between PC advancement and hub gene expression.

Promoter methylation is essential in controlling gene expression in normal and cancer cells. In this study, we noticed that the promoter regions of five HGs, such as KRT19, MMP1, COL11A1, SDC1, FN1, and COL5A1 were highly methylated in normal compared to tumor patients. This result suggests that losing promoter methylation in PC is responsible for the higher expression of these six HGs. In a recent investigation addressing tamoxifen resistance in breast cancer, elevated expression of MMP1 was identified as a resistance‐driving gene associated with promoter methylation.^72^ Genetic alteration of our HGs might contribute to abnormal expression that results in PC initiation and progression.

Network analysis revealed eight transcription factors including FOXC1, GATA2, FOXL1, CREB1, YY1, STAT3, TFAP2A, and SREBF1 having high degree interaction with HGs. FOXC1 is an oncogenic transcription factor that is upregulated in PC and promotes PC growth and metastasis through IGF‐1R signaling.^73^ In addition, a study conducted by Sun et al. reported higher expression of SREBF1 in PC, which has a role in tumor progression and can be used as a prognostic biomarker for PC.^74^ On the other hand, miRNAs network analysis revealed three highly connected miRNAs (hsa‐mir‐145‐5p, hsa‐mir‐27a‐3p, and hsa‐mir‐124‐3p) that are downregulated in PC. The lower expression of these miRNAs may not silence gene expression of our HGs at the post‐transcriptional level. miRNAs are non‐coding RNA molecule that modulates gene expression by selectively binding to specific sequences within the mRNA of target genes This binding can exert two effects: it can either inhibit the translation of mRNA into protein or induce its degradation. In hepatocellular carcinoma, hsa‐mir‐145‐5p is downregulated and its downregulation is associated with tumor progression.^75^ In addition, the expression of hsa‐mir‐145‐5p is reduced in breast cancer, and this decrease is linked to larger tumor size, distal metastasis, and shorter overall survival.^76^

Survival analysis was performed to determine the prognostic value of HGs, revealing a trend where PC patients exhibiting elevated expression of KRT19, MMP1, COL11A1, SDC1, ITGA2, POSTN, and FN1 experienced shorter overall survival. Moreover, relapse‐free survival was negatively correlated with hub gene expression, indicating that upregulation of HGs is related to lower relapse‐free survival of PC patients. We performed ROC analysis to determine whether our HGs can be used as a diagnostic and prognostic biomarker. Notably, our findings indicated that these HGs exhibit promise as excellent candidates for both diagnostic (AUC > 0.75) and prognostic (AUC > 0.64) biomarkers in PC. In a wet laboratory investigation conducted by Steffen Deichmann et al., it was concluded that the overexpression of ITGA2 was linked to reduced survival in PC, aligning with the outcomes observed in our current study.^77^ In addition, high plasma MMP1 concentration correlates with a poor colon cancer prognosis.^78^ On the other hand, several studies based on bioinformatics have revealed a lower survival probability of breast cancer, gastric cancer, mesothelioma, and colorectal cancer patients with higher expression of COL1A1, COL1A2, and COL11A1.^79^, ^80^, ^81^, ^82^

The tumor microenvironment is a complex master driver for tumor growth, invasion, and metastasis. Along with other components, immune cell infiltration is a major contributor to tumor microenvironment. In this study, we observed the positive correlation of T‐cell CD8+, neutrophils, monocytes, macrophages, dendritic cells, and NK cells with the higher expression of COL1A1, COL1A2, COL5A1, COL11A1, FN1, and POSTN but negative correlation with KRT19, MMP1, ITGA2, and SDC1. Immune cell infiltration can recognize and kill the cancer cells which is positively correlated with overall disease survival in many cancers.^83^ However, in this present study, most HGs' expressions are positively associated with immune cell infiltration but negatively correlated with overall and relapse‐free survival. This might happen due to cancer heterogeneity, immune suppressive tumor microenvironment, and lymphocyte ineffectiveness against outnumbered cancer cells. It has been demonstrated that CD8+ T cells initially express CD49b, then acquire CD49a as they migrate into the TME. However, during tumor progression, CD49b expression decreases, resulting in dysfunction of CD8+ T cells.^84^ Our study suggests that immunotherapy alone may not benefit patients with higher expression of these HGs. Combining immune therapies with chemotherapy and radiation therapy is imperative in this aspect. Therefore, the HGs and associated tumor‐infiltering immune cells can be biomarkers for predicting treatment response and survival in distinct patient subgroups. Since we assume that immunotherapies alone might not work against our HGs, we found nine drugs that can downregulate our HGs. Out of the nine compounds analyzed, we identified two steroid hormones: Estradiol and Progesterone. Studies have found that using Estradiol and Progesterone hormone might reduce the risk of PC.^85^, ^86^

However, our study presents certain limitations. Initially, the identification of HGs relied on three datasets, and the inclusion of a greater number of datasets could enhance the robustness of the evidence. Additionally, the majority of our HGs are linked to the ECM, leaving unanswered questions regarding the specific mechanisms by which these HGs remodel the ECM to facilitate PC progression. Furthermore, a notable flaw in our study is the absence of in vitro and in vivo validation. As a result, our future research endeavors will seek to validate the outcomes of this meta‐analysis through the incorporation of wet laboratory approaches.

## 5 CONCLUSION

This study has identified 10 ECM‐related HGs through integrated bioinformatics approaches. The identification of these HGs provides potential diagnostic biomarkers that can be used for early detection of PC. Moreover, the elevated expression of those HGs is correlated with lower survival among PC patients, indicating their potential as prognostic markers. Furthermore, this study also explored chemical compounds with substantial interaction profiles that could serve as effective therapeutic agents, expanding the potential treatment options for PC. Overall, the results of our study provide valuable information that can be further explored and potentially translated into clinical applications for the diagnosis, prognosis, and treatment of PC. However, the study has limitations, such as being based on a limited number of datasets and lacking in vitro and in vivo validation. Further research is recommended to validate the findings using wet laboratory approaches.

## AUTHOR CONTRIBUTIONS

**Md Roman Mogal:** Conceptualization (equal); formal analysis (lead); investigation (equal); methodology (lead); project administration (supporting); software (lead); supervision (supporting); visualization (lead); writing – original draft (lead); writing – review and editing (supporting). **Jasmin Akter Jame:** Formal analysis (supporting); visualization (supporting); writing – original draft (supporting). **Md Sohel:** Data curation (equal); formal analysis (supporting); writing – review and editing (supporting). **Md Mozibullah:** Data curation (equal); formal analysis (supporting); writing – review and editing (supporting). **Md Rashel Mahmod:** Formal analysis (supporting); software (supporting). **Asadullah Junayed:** Formal analysis (supporting); software (supporting). **Newton Kar:** Formal analysis (supporting); software (supporting). **Lubatul Arbia:** Formal analysis (supporting); visualization (supporting). **Md Abdullah Al Mamun:** Project administration (supporting); supervision (supporting); writing – review and editing (supporting). **Md Asaduzzaman Sikder:** Conceptualization (equal); investigation (equal); methodology (supporting); project administration (lead); supervision (lead); writing – review and editing (lead).

## FUNDING INFORMATION

This present study did not obtain any specific grant from funding organizations in the public, private, or nonprofit sectors.

## CONFLICT OF INTEREST STATEMENT

The authors have stated explicitly that there are no conflicts of interest in connection with this article.

## ETHICS STATEMENT

Not applicable.

## Supporting information


**Figure S1:** Examining the expression of hub genes in pancreatic cancer using the OncoDB database. The red bar graph illustrates cancerous tissue, while the black graph depicts normal tissue samples. *P* > 0.001 is considered statistically significant.

**Figure S2:** Protein expression of hub genes in pancreatic cancer based on tumor grade. Different bar graph color indicates different tumor grade.

**Figure S3:** Promoter methylation status of hub genes in pancreatic cancer. The blue bar graph represents nontumor tissue, while the red graph represents tumor tissue. P > 0.05 is considered statistically significant.

**Figure S4:** Expression status of three miRNA (has‐mir‐145, has‐mir‐27a and has‐mir‐124‐2) associated with hub genes. The blue bar graph illustrates normal tissue, while the red graph depicts tumor tissue samples.

**Figure S5:** ROC curve analysis for hub genes expression.

**Figure S6:** ROC curve analysis for survival data of hub genes.

**Figure S7:** Structural view of nine compounds that might act as potential therapeutics targeting hub genes.

**Table S1:** Correlation analysis between HGs and immune cells infiltration using TISIDB.

## Data Availability

All data or information used in this study is available upon reasonable request from the corresponding author.
