# Supplementary figures and images for: Integrated bioinformatics analysis reveals upregulated extracellular matrix hub genes in pancreatic cancer: Implications for diagnosis, prognosis, immune infiltration, and therapeutic strategies
Source: Cancer Rep (Hoboken). 2024 Apr 19;7(4):e2059. doi: 10.1002/cnr2.2059 (PMC11027013; doi:10.1002/cnr2.2059)

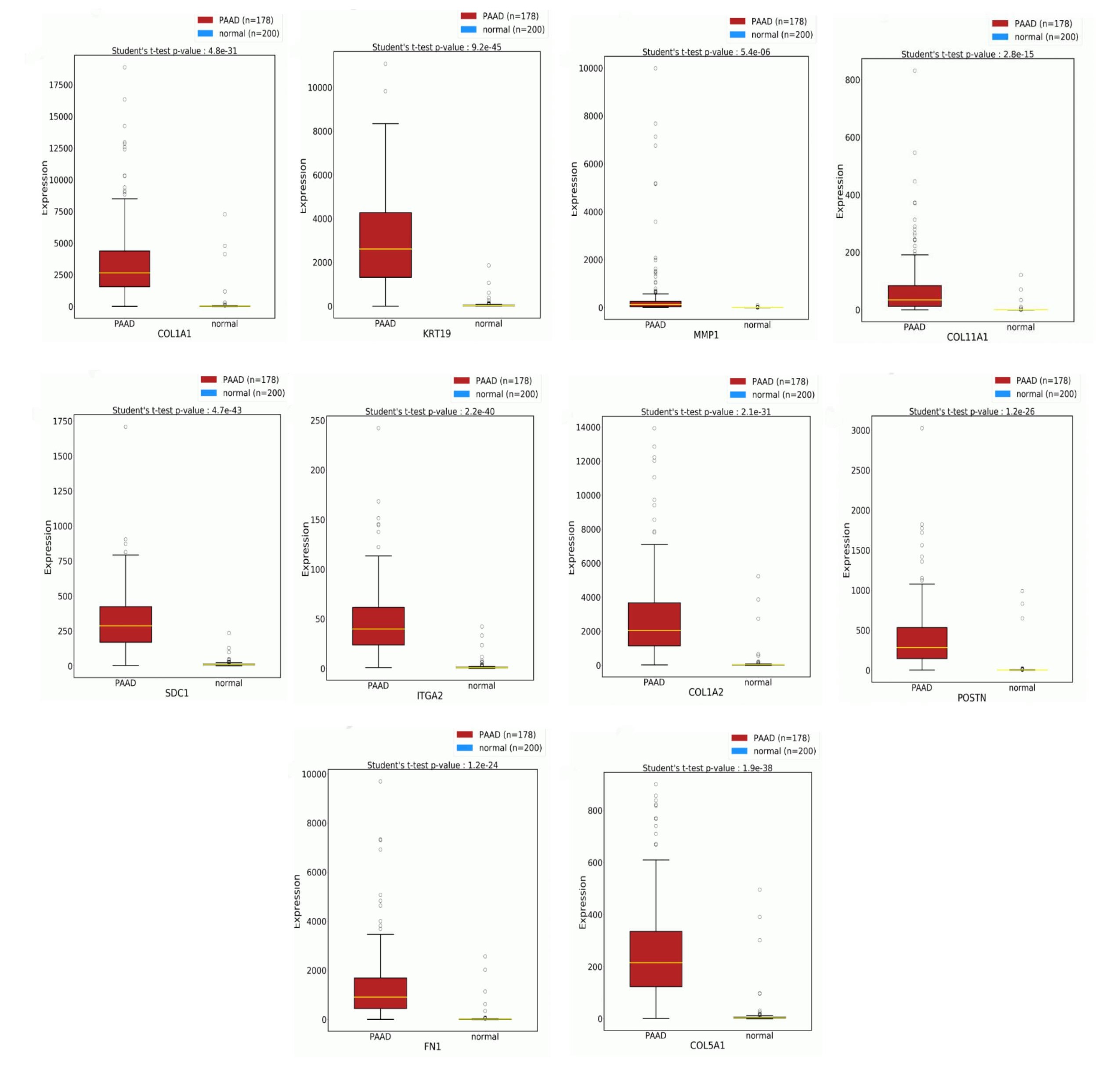

Supplement: Supplementary file 1 — Figure S1: Examining the expression of hub genes in pancreatic cancer using the OncoDB database. The red bar graph illustrates cancerous tissue, while the black graph depicts normal tissue samples. P > 0.001 is considered statistically significant. [file CNR2-7-e2059-s006.jpg]

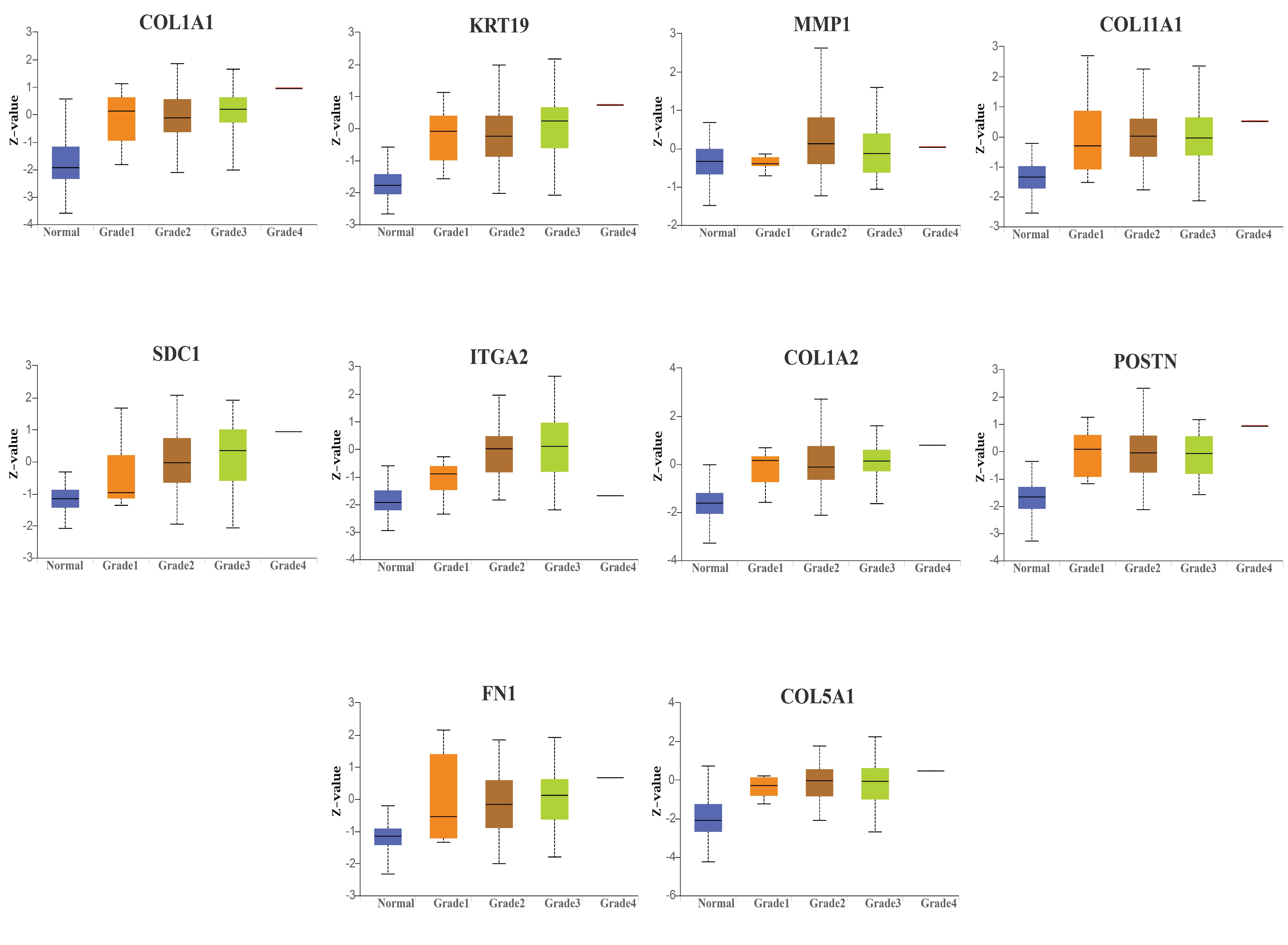

Supplement: Supplementary file 2 — Figure S2: Protein expression of hub genes in pancreatic cancer based on tumor grade. Different bar graph color indicates different tumor grade. [file CNR2-7-e2059-s004.jpg]

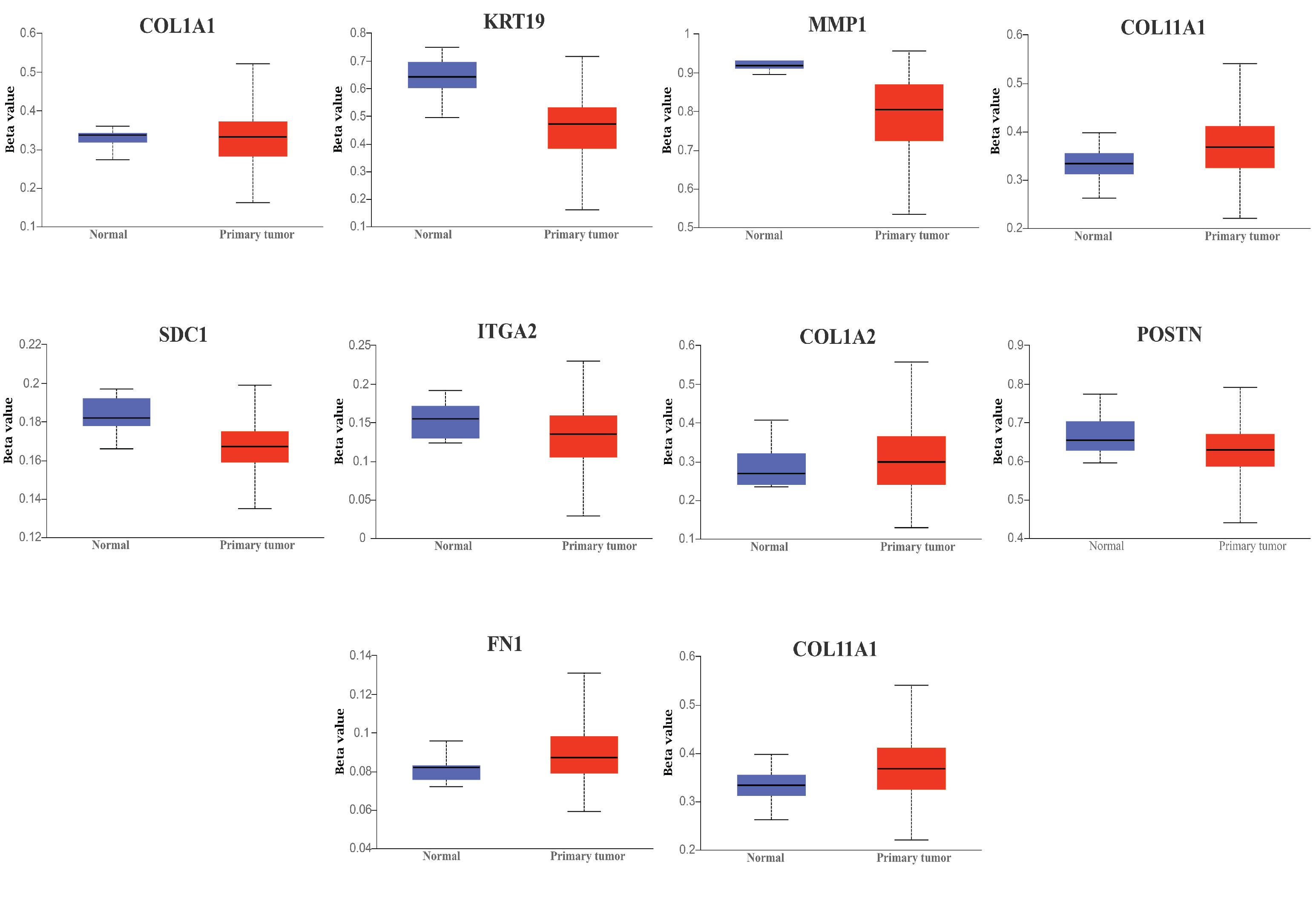

Supplement: Supplementary file 3 — Figure S3: Promoter methylation status of hub genes in pancreatic cancer. The blue bar graph represents nontumor tissue, while the red graph represents tumor tissue. P > 0.05 is considered statistically significant. [file CNR2-7-e2059-s002.jpg]

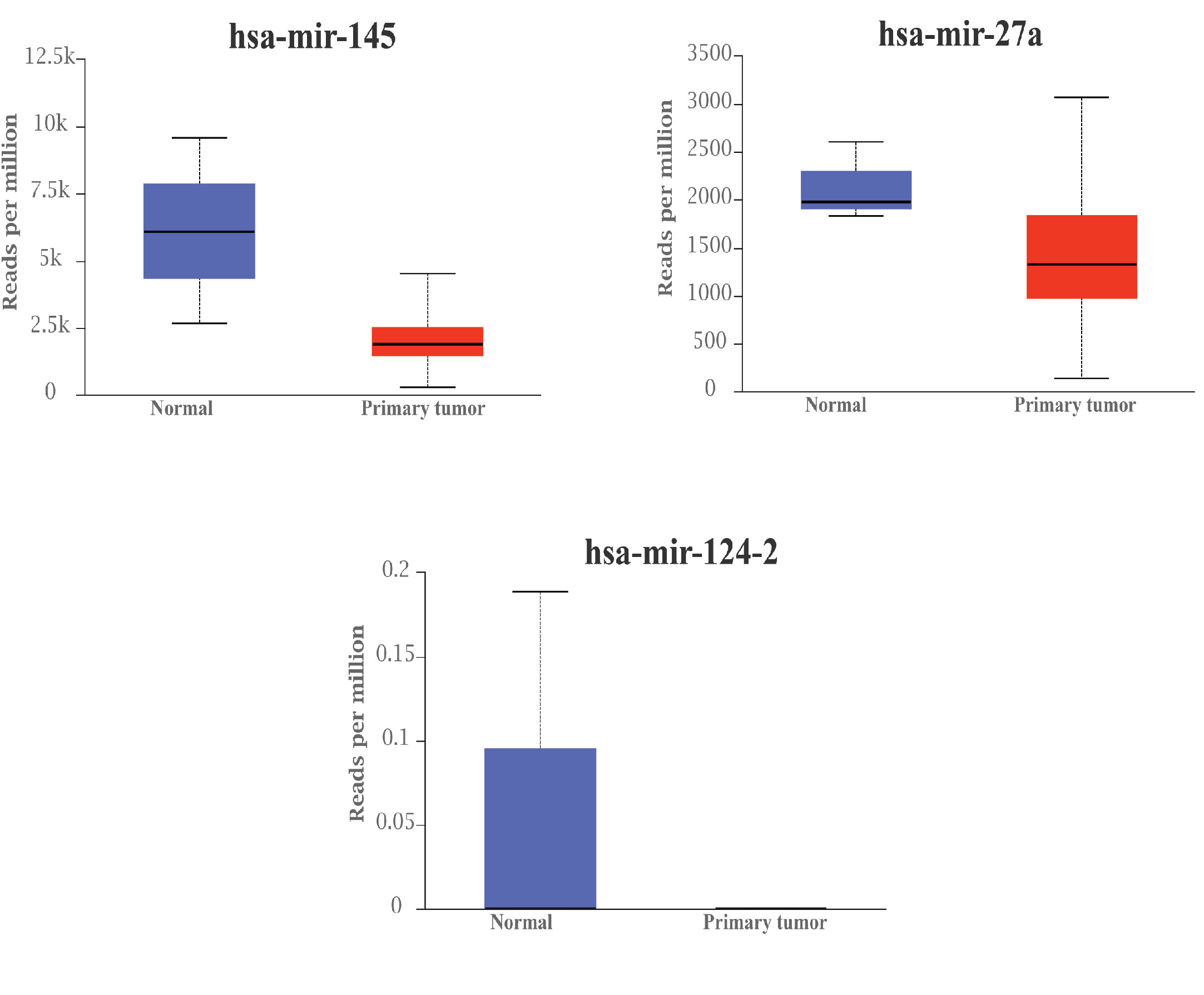

Supplement: Supplementary file 4 — Figure S4: Expression status of three miRNA (has‐mir‐145, has‐mir‐27a and has‐mir‐124‐2) associated with hub genes. The blue bar graph illustrates normal tissue, while the red graph depicts tumor tissue samples. [file CNR2-7-e2059-s008.jpg]

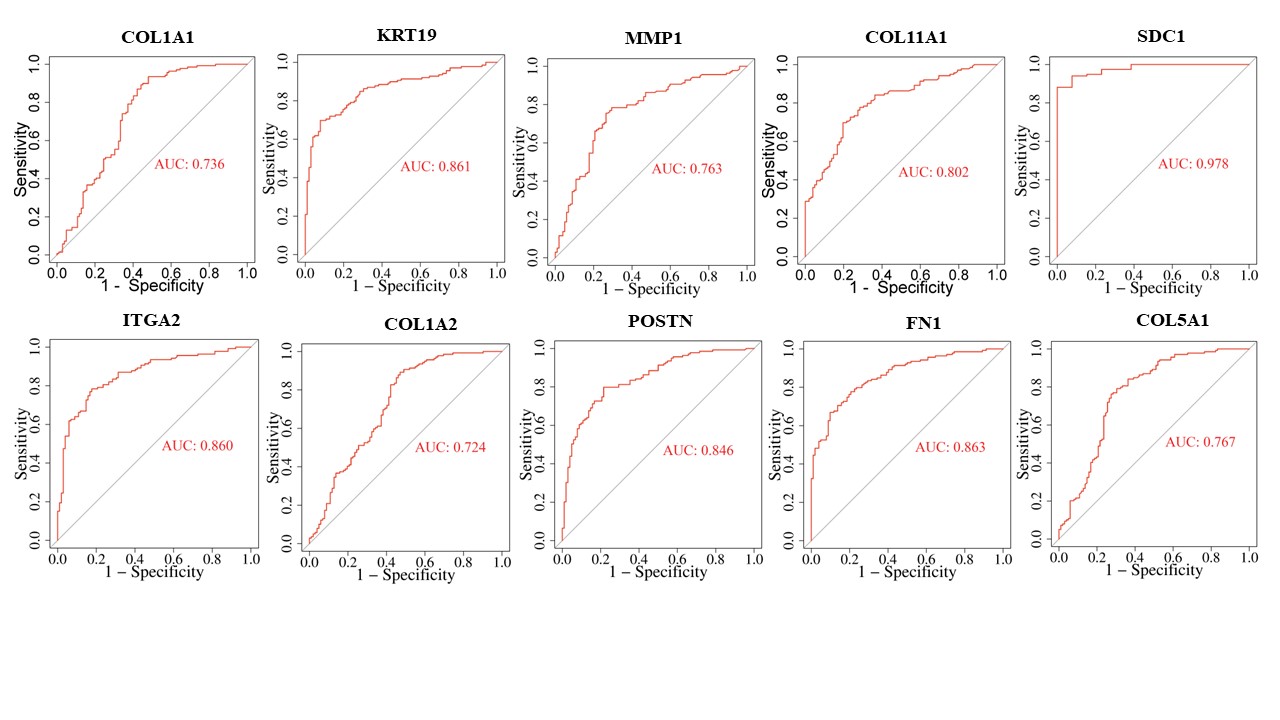

Supplement: Supplementary file 5 — Figure S5: ROC curve analysis for hub genes expression. [file CNR2-7-e2059-s007.jpg]

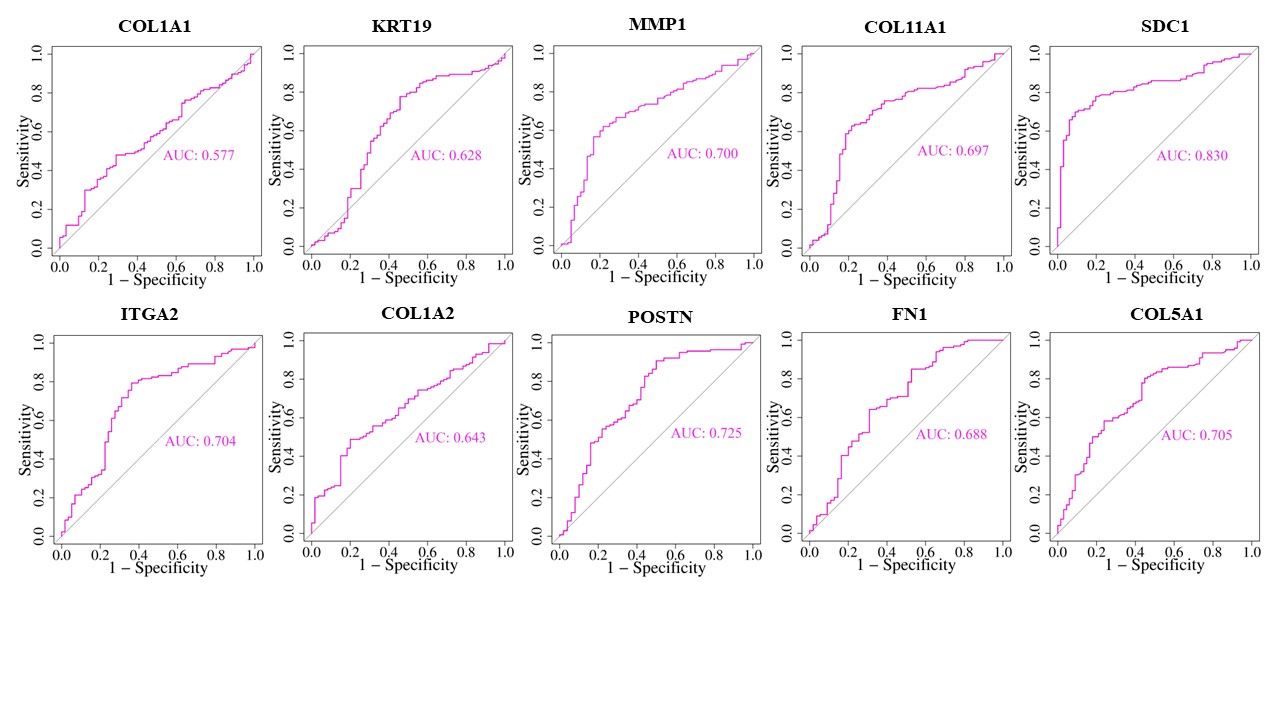

Supplement: Supplementary file 6 — Figure S6: ROC curve analysis for survival data of hub genes. [file CNR2-7-e2059-s001.jpg]

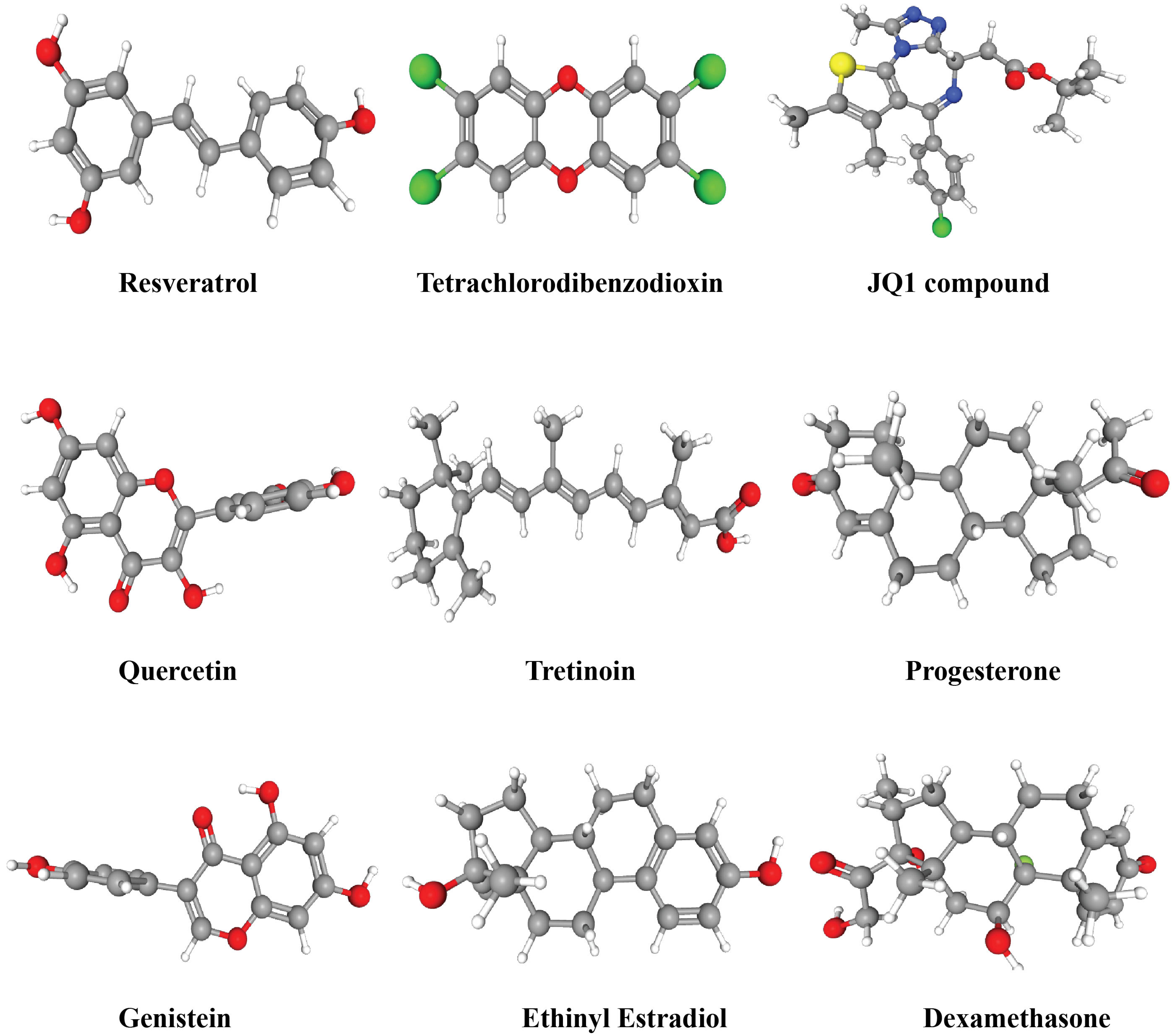

Supplement: Supplementary file 7 — Figure S7: Structural view of nine compounds that might act as potential therapeutics targeting hub genes. [file CNR2-7-e2059-s003.jpg]
