# Supplementary material for: Integrated bioinformatics analysis reveals upregulated extracellular matrix hub genes in pancreatic cancer: Implications for diagnosis, prognosis, immune infiltration, and therapeutic strategies
Source: Cancer Rep (Hoboken). 2024 Apr 19;7(4):e2059. doi: 10.1002/cnr2.2059 (PMC11027013; doi:10.1002/cnr2.2059)
Supplement: Supplementary file 8 — Table S1: Correlation analysis between HGs and immune cells infiltration using TISIDB. [file CNR2-7-e2059-s005.docx]

| Genes | CD8+ | | Neutrophil | | Dendritic cells | | Macrophage | | NK cells | |
| --- | --- | --- | --- | --- | --- | --- | --- | --- | --- | --- |
|  | Cor | *P* | Cor | *P* | Cor | *P* | Cor | *P* | Cor | *P* |
| COL1A1 | 0.4150 | *** | 0.188 | *** | 0.3980 | *** | 0.406 | *** | 0.567 | *** |
| COL1A2 | 0.501 | *** | 0.207 | ** | 0.419 | *** | 0.451 | *** | 0.643 | *** |
| COL5A1 | 0.285 | *** | 0.154 | * | 0.413 | *** | 0.367 | *** | 0.575 | *** |
| COL11A1 | 0.219 | ** | 0.097 | 0.2 | 0.412 | *** | 0.272 | *** | 0.461 | *** |
| FN1 | 0.249 | ** | 0.158 | * | 0.451 | *** | 0.323 | *** | 0.554 | *** |
| POSTN | 0.242 | ** | 0.204 | * | 0.456 | *** | 0.338 | *** | 0.509 | *** |
| KRT19 | 0.077 | 0.3 | -0.167 | * | 0.142 | 0.1 | -0.269 | *** | -0.185 | * |
| MMP1 | 0.05 | 0.5 | 0.072 | 0.3 | 0.074 | 0.3 | 0.086 | 0.2 | 0.065 | 0.4 |
| ITGA2 | -0.091 | 0.2 | -0.007 | 0.9 | 0.176 | * | -0.155 | * | 0.132 | 0.1 |

**Table S1:** Correlation analysis between HGs and immune cells infiltration using TISIDB

*P < 0.05; **P < 0.01; ***P < 0.0001
